# Family Members Dealing With Childhood Cancer: A Study on the Role of Family Functioning and Cancer Appraisal

**DOI:** 10.3389/fpsyg.2019.01405

**Published:** 2019-06-19

**Authors:** Marieke Van Schoors, Annick Lena De Paepe, Koenraad Norga, Veerle Cosyns, Hanne Morren, Trui Vercruysse, Liesbet Goubert, Lesley Liliane Verhofstadt

**Affiliations:** ^1^Department of Experimental Clinical and Health Psychology, Ghent University, Ghent, Belgium; ^2^Department of Pediatric Oncology, Antwerp University Hospital, Antwerp, Belgium; ^3^Department of Pediatric Oncology, University Hospital Brussels, Brussels, Belgium; ^4^Department of Pediatric Hematology-Oncology and Stem Cell Transplantation, Ghent University Hospital, Ghent, Belgium; ^5^Department of Pediatrics, University Hospitals Leuven, Leuven, Belgium

**Keywords:** families, pediatric cancer, family functioning, cancer appraisal, individual adjustment

## Abstract

**Objectives:**

Childhood cancer is a life-threatening disease that poses significant challenges to the life of the diagnosed child and his/her family members. Based on the ABCX-model, the aim of the current study was to explore the association between family functioning, cancer appraisal and the individual adjustment of patients, parents and siblings.

**Methods:**

Participants were 60 children with leukemia or non-Hodgkin lymphoma, 172 parents and 78 siblings (115 families). Time since diagnosis varied from zero to 33 months. Patients, parents and siblings completed the Family Environment Scale (FES), Perceived Stress Scale, Situation-Specific Emotional Reactions Questionnaire and Pediatric Quality of Life Inventory/Maudsley Marital Questionnaire.

**Results:**

Family functioning and the appraisal of the cancer diagnosis proved to be related to patients’, parents’ and siblings’ cancer-related emotions and quality of life post-diagnosis. In addition, family members differed in their perception of some family functioning domains, the appraisal of the cancer diagnosis, positive feelings and quality of life.

**Discussion:**

Our findings led to the conclusion that family functioning and the appraisal of the cancer diagnosis are important for the individual adjustment of patients, parents and siblings when facing a diagnosis of cancer in the child. Differences across members within one family and differences between families speak to the need of screening all family members and intervening at the level of individual as well as the family unit.

## Introduction

Every year, approximately 300,000 children are diagnosed with cancer worldwide (Steliarova-Foucher et al., 2017). Although there has been a huge improvement in survival rates in the last decades – with currently a 5-year survival rate of 83.9% (National Cancer Institute, 2014) – the psychosocial impact of childhood cancer cannot be underestimated. Children diagnosed with cancer are often confronted with social and/or emotional problems during or after treatment (Kazak et al., 2001; Michel et al., 2010; Brinkman et al., 2016). Previous studies also revealed that the turmoil and disruption created by childhood cancer reach beyond the diagnosed child and impact the parents and possible siblings as well (Kazak et al., 2001; Kestler and LoBiondo-Wood, 2012; Van Schoors et al., 2017). More specifically, parents often report feelings of posttraumatic stress, uncertainty, anxiety and depression, especially shortly after diagnosis (Patino-Fernandez et al., 2008; Vrijmoet-Wiersma et al., 2008). In addition, some siblings show increased symptoms of post-traumatic stress, negative emotional reactions and poor quality of life when compared to norms or control groups (Alderfer et al., 2010; Long et al., 2018).

**FIGURE 1:**
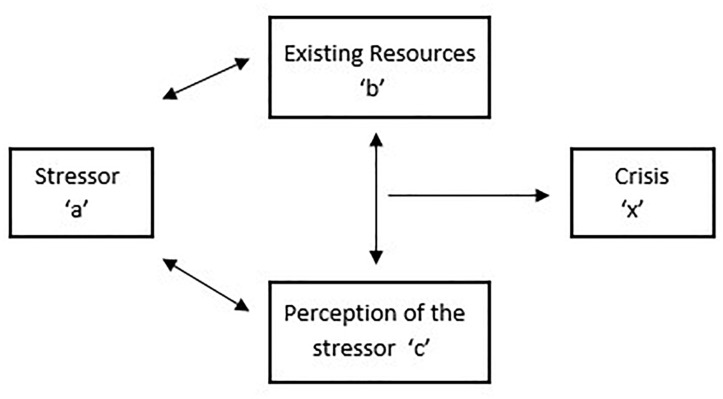
ABCX model (Hill, 1958).

It should be noted, however, that the research literature on the individual adjustment of children diagnosed with cancer and their family members documents a considerable variability in outcomes: while most show resiliency, some report adjustment problems after diagnosis. This idea of variability in adjustment to stressors is a key principle of the so-called ABCX-model (Hill, 1958; Figure 1), one of the major family-stress models (Weber, 2011). This model assumes that a stressor (“a”) interacts with the family members’ crisis-meeting resources (“b”) and the appraisal (“c”) family members make of the stressful event, and that this interaction produces the amount of crisis or maladjustment (“x”) in each family member (Weber, 2011). In other words, how an individual (the ill child and his/her family members) responds to or deals with childhood cancer is the result of an interaction between his/her available resources and his/her perception of the illness: the more resources and the more one perceives the illness as manageable instead of uncontrollable, the better the individual adjustment. Resources can be interpreted as factors that, by their presence, keep the individual from crisis or, by their absence, urges a family member into crisis. Resources can be situated at three levels: the individual level (e.g., personality; Erickson and Steiner, 2001), the family level (e.g., family functioning; Van Schoors et al., 2017) and the contextual level (e.g., network support; Corey et al., 2008).

Existing research on the individual adjustment of children diagnosed with cancer and their family members is limited in three ways. First, most research is a-theoretical (i.e., not based on a theoretical framework; Van Schoors et al., 2015), so the selection of the variables within studies (type and their role) and the interpretation of the results is rather arbitrary. Second, up till now, most of the research that tried to explain why some family members adjust better than others after a diagnosis of childhood cancer focused on individual and contextual resources, and less research attention has been paid to *family resources*. However, the way in which the family as a whole deals with and responds to childhood cancer (“family functioning”) is generally assumed to impact the adjustment of all members within the family (e.g., Van Schoors et al., 2017). Indeed, when facing childhood cancer, family members need to cope with intense emotions, communicate effectively and renegotiate roles and responsibilities to accommodate the demands of treatment (Kazak et al., 2004; Marcus, 2012; Van Schoors et al., 2015), and poorly functioning families who struggle with these demands may be at greater risk for adjustment problems (e.g., Long et al., 2013; Myers et al., 2014; Van Schoors et al., 2017). Third, within the childhood cancer literature, most studies only include one single respondent (e.g., Van Schoors et al., 2015), rather than considering the perspectives of *all* family members. As a consequence, the interdependence between family members and the bidirectional relationships within families are, to date, mostly neglected.

Addressing these three limitations, the aim of the present study was 2-fold. First, relying on the ABCX model as theoretical framework, we aimed to investigate how the interplay of family functioning (a key family resource; “b”) and the appraisal of the cancer diagnosis (perception/definition; “c”) predicts cancer-related emotional well-being and perceived quality of life (individual adjustment, “x”) in patients, parents and siblings when facing childhood cancer. More specifically, we expected that better family functioning and perceiving the illness as more manageable and less uncontrollable, as well as the interplay between both, will be associated with better individual outcomes (i.e., less negative cancer-related emotions, more positive cancer-related emotions and better quality of life) in patients, parents and siblings. The secondary aim was more explorative in nature and concerns the investigation of similarities and differences in the appraisal of the cancer diagnosis, the perception of family functioning, cancer-related emotions and perceived quality of life across members within one family.

## Materials and Methods

### Participants

The sample consisted of 115 families where one child has been diagnosed with leukemia or non-Hodgkin lymphoma. All families were Caucasian and living in the Flemish part of Belgium. Across the families, time since diagnosis varied from 0 to 33 months (*M* = 6,90, *SD* = 8,05). The ill child’s mean age was 6,60 (*SD* = 4,84; Range = 0–19). In 24 families (21%), the diagnosed child was the only child. The remaining families had either two (52 families; 45%), three (28 families; 24%), four (9 families; 8%) or five (2 families; 2%) children.

Due to the questionnaires’ age limits (e.g., the Family Environment Scale (FES) is only applicable for children aged 11 and above) and the willingness of the different family members to participate, data from 60 ill children, 172 parents and 78 siblings were included in the present study. More details on the sample are listed in Table 1. Ethical approval from the University Hospitals of Ghent, Brussels, Antwerp, and Louvain had been secured for the study. Written informed consent forms were obtained from all the participating parents in this study, as well as all the participating children above the age of 12. Parental consent was obtained for all participating children under the age of 16.

**Table 1:** Background characteristics of the study sample.

		Demographic variable		
Families		*N*		115
		Age ill child, mean (*SD*)		6,60 (4,84)
		Sex ill child, boys, *n (%)*		69 (60%)
		Diagnosis, *n (%)*	Acute lymphoblastic leukemia (ALL)	85 (73,9%)
			Acute myeloid leukemia (AML)	8 (7%)
			Chronic myeloid leukemia (CML)	2 (1,7%)
			Non-Hodgkin lymphoma	20 (17,4%)
		Time since diagnosis in months (*SD*; Range)		6,90 (8,05; 0–33)
		Family status, *n (%)*	Married/Co-habiting	100 (87%)
			Divorced	8 (7%)
			Single parent	3 (3%)
			Stepfamily	4 (3%)
Participating	Ill child	N		60
Family members^1^		Sex, boys, *n (%)*		34 (56,7%)
		Age, mean (*SD*)		9,90 (3,76)
	Parents	N		172
		Sex, men, *n (%)*		73 (42%)
		Age, mothers mean (*SD*)		37,58 (6,31)
		Age, fathers mean *(SD)*		40,18 (6,46)
	Siblings	N		78
		Sex, boys, *n (%)*		37 (47,4%)
		Age, mean (*SD; range*)		10,82 (4,92; 5–25)


*^*1*^Note that only the characteristics of the participating family members are summarized.*

### Procedure

The current study is part of a larger ongoing study examining the impact of childhood cancer on families, i.e., “UGhent Families and Childhood Cancer study.” For this large-scale study, families of children diagnosed with leukemia or non-Hodgkin lymphoma between the age of zero and 18 years at the moment of diagnosis were invited to take part in a longitudinal survey study. Specifically, all children (patients and siblings) aged 5 years and more and both parents were asked to complete a set of questionnaires at five different time points (diagnosis to 2.5 years post-diagnosis). For this study, only the first measurement of all family members was included. Exclusion criteria for participation were: (1) not speaking Dutch (*N* = 20), (2) expression of a developmental disorder in the diagnosed child (*N* = 9), and (3) relapse (*N* = 6). Over a period of 4 years, 115 families participated (56% of the eligible families). The most important reasons for non-participation were being overwhelmed by the diagnosis and lack of time.

### Measures

Patients, parents and siblings separately filled out a similar set of questionnaires, as described below. However, due to a minimum age limit of the questionnaires, some younger children did not complete all questionnaires. For each questionnaire, the minimum age and the number of participants excluded for the questionnaire based on this minimum age (“N_age_”) are reported.

#### Family Functioning

The Dutch version of the Family Environment Scale (FES; Moos and Moos, 1994) was used to evaluate family functioning. The questionnaire contains 77 “yes–no” items, distributed across seven subscales: (1) cohesion (e.g., “we support each other anyway”), (2) expressiveness (e.g., “we have many spontaneous conversations in our family”), (3) conflict (e.g., “we quarrel a lot at home”), (4) organization (e.g., “we take care that our rooms are cleaned up”), (5) control (e.g., “we pay close attention to being at home on time”), (6) norms and values (e.g., “working first than playing is a rule in our family”), and (7) social orientation (e.g., “everyone has hobbies in our family”). Two composite scores can be calculated as well: the family relation index (FRI) (cohesion + expressiveness - conflict) and the family structure index (FSI) (organization + control), reflecting the affective nature of the family relationships and the extent to which the family is structured and open to change, respectively. Higher FES composite scores reflect higher emotional closeness within the family (FRI; more cohesion and expressiveness and less conflict) and a more rigid family structure (FSI; more control and organization). The FES is applicable for children aged 11 and above (*N*_age_ = 82; 37 patients, 45 siblings), and has good reliability and validity (Jansma and De Coole, 1995). In the present study, the overall Cronbach’s alpha reliabilities ranged from 0.71 (fathers) to 0.76 (siblings) for the relation index and from 0.57 (mothers) to 0.67 (siblings) for the structure index. The low Cronbach’s alphas for the FSI subscale could not be improved by dropping one or more items.

#### Appraisal of the Cancer Diagnosis

The Perceived Stress Scale (PSS; Cohen et al., 1983) measures the extent to which a person perceives the last month as unpredictable, uncontrollable, and overloading. For this study, the instruction of the questionnaire was adapted and the participant was asked to rate the extent to which s/he perceives her/his *life since the cancer diagnosis* as unpredictable, uncontrollable, and overloading. The questionnaire consists of 10 items, rated on a 5-point Likert scale from 0 (never) to 4 (very often). Total scores range from 0 to 40 and higher scores indicate perceiving the illness as more uncontrollable and less manageable. An example item is “since the cancer diagnosis, how often did you feel that things were going as you wanted?” The PSS is applicable for children aged 10 and above (*N*_age_ = 71; 32 patients, 39 siblings), and has good reliability (e.g., Golden-Kreutz et al., 2005). In addition, 3 participants older than 10 years (1 patient and 2 siblings) did not complete the questionnaire. In the present study, Cronbach’s alpha coefficients were 0.74, 0.54, 0.51, and 0.63, for patients, mothers, fathers and siblings, respectively. The low Cronbach’s alphas for the mothers and the fathers could not be improved by dropping one or more items.

#### Cancer-Related Emotions

The Situation-Specific Emotional Reactions Questionnaire (SSERQ; Grootenhuis and Last, 1997; Houtzager et al., 2004) is developed to assess emotional reactions in families where one child has been diagnosed with cancer. Different versions are available for patients (30 items), parents (30 items) and siblings (26 items), but all are divided in four subscales: (1) loneliness (e.g., “I feel lonely”), (2) uncertainty (e.g., “I am afraid to lose my child”), (3) positive feelings (e.g., “I am proud that I persevere”), and, (4) helplessness/emotional involvement (e.g., “I regret that my parents have to undergo this”). This latter subscale is called “helplessness” in the patients’ and parents’ version, and “emotional involvement” in the siblings’ version. However, given the consensus on a content level, and in agreement with the authors of the subscales, this subscale will further be referred to as “helplessness.” All items are rated on a 4-point Likert scale from 0 (almost never) to 3 (almost always). The higher the scores, the more emotional reactions, both negative (loneliness, uncertainty and helplessness) and positive (positive feelings). The questionnaire is applicable from the age of 7 (*N*_age_ = 24; 13 patients, 11 siblings) and has satisfactory to good validity and reliability (Grootenhuis and Last, 1997; Houtzager et al., 2004). In addition, 5 participants older than 7 years (1 patient and 4 siblings) did not complete the questionnaire. In the present study, Cronbach’s alpha coefficients ranged from 0.77 (patients) to 0.88 (siblings) for uncertainty, from 0.68 (patients) to 0.88 (siblings) for helplessness, from 0.67 (patients) to 0.92 (mothers) for loneliness and from 0.58 (siblings) to 0.81 (fathers) for positive emotions.

#### Quality of Life (QoL)

The pediatric quality of life inventory (PedsQL) and the general life satisfaction subscale of the Maudsley Marital Questionnaire (MMQ) were used to assess quality of life in children and parents, respectively. The PedsQL (Varni et al., 1999) measures children’s health-related quality of life. Different versions of the questionnaire are available, for example, the PedsQL^TM^ 3.0 Cancer Module (children diagnosed with cancer) and PedsQL^TM^ Generic Core Scales (healthy children). In this study, the PedsQL^TM^ 3.0 Cancer Module measured the diagnosed child’s quality of life and is composed of 27 items comprising eight dimensions: (1) Pain and Hurt (e.g., “I have a lot of pain”), (2) Nausea (e.g., “I feel too nauseous to eat”), (3) Procedural Anxiety (e.g., “I get scared when blood has to be taken”), (4) Treatment Anxiety (e.g., “I get scared when I have to go to the doctor”), (5) Worry (e.g., “I worry about the side effects of the medical treatments”), (6) Cognitive Problems (e.g., “I have trouble remembering what I read”), (7) Perceived Physical Appearance (e.g., “I am ashamed when others see my body”), and (8) Communication (e.g., “it’s difficult to ask nurses and doctors questions”). The PedsQL^TM^ Generic Core Scales measured the siblings’ quality of life and is composed of 23 items comprising four dimensions: (1) Physical functioning (e.g., “it’s hard for me to run”), (2) Emotional functioning (e.g., “I feel angry”), (3) Social functioning (e.g., “other kids tease me”), and (4) School functioning (e.g., “I forget things”). Within both questionnaires, all items are scored on a five-point Likert-scale (0 = never to 4 = almost always). Each of the item scores is reversed and rescaled to a 0–100 scale: a score of 100 represents the best quality of life possible, a score of 0 the worst quality of life possible. Scale scores, as well as the sum score, are computed by adding together the different item scores and dividing this obtained score by the number of items answered. The questionnaire is applicable from the age of 5 (*N*_age_ = 0) and has sufficient to good validity and reliability (Varni et al., 2001). Five participants older than 5 years (1 patient and 4 siblings) did not complete the questionnaire. In the present study, Cronbach’s alpha coefficients were 0.89 and 0.89, for patients and siblings, respectively.

The Maudsley Marital Questionnaire (MMQ; Arrindell et al., 1983) evaluates life in general (e.g., “Are you competent and successful at your job and your housework?”) and the marital/sexual relationship (e.g., “How much are you committed to this marriage?”). The MMQ contains 20 items, each of which is rated on a 0–8 scale, with 0 representing the optimum response. Higher scores indicate more maladjustment. The MMQ has good reliability and validity and the psychometric qualities of the Dutch version were also found to be satisfactory (Arrindell et al., 1983; Orathinkel et al., 2007). In the present study, the MMQ was not completed by single or divorced parents (*N* = 15; 9 mothers and 6 fathers) and only the subscale measuring general life satisfaction (i.e., satisfaction with life, household and social network) was taken into account, with a Cronbach’s alpha reliability of 0.70 (mothers) and 0.72 (fathers).

Parents’ scores on the MMQ were reversed, so for all participants (patients, siblings, mothers, fathers) higher scores (on the PedsQL and the MMQ, respectively) indicate better quality of life.

### Data Analytic Strategy

A multilevel (or hierarchically nested) approach was used to structure the data. This means that observations at one level of analysis (individual family members) were nested within another level of analysis (family). Multilevel modeling was preferred over ordinary-least-squares (OLS) methods, such as ANOVA, because it provides better parameter estimates with nested data (Kenny et al., 1998). The R-package *lme4* (Bates et al., 2015) was used to analyze multilevel data. The amount of variance attributable to each of the grouping structures were calculated using the function *icc* of the R package *sjstats* (Lüdecke, 2019). Continuous predictor variables were centered around their mean value to improve interpretability of the regression coefficients (Schielzeth, 2010).

To investigate whether family functioning and the appraisal of the cancer diagnosis affect *cancer-related emotions* and perceived *QoL*, separate models were fitted with SSERQ scores and the QoL score, respectively, as outcome variables. For *cancer-related emotions* four separate models were fitted for the subscales of the questionnaire (loneliness, uncertainty, positive emotions and helplessness). For *QoL* two separate models were fitted, one for the mothers and fathers (with scores on the MMQ as outcome variable) and one for the patients and siblings (with scores on the PedsQL as outcome variable). Predictor variables of interest were FES scores as a measure of *family functioning* and the PSS score as a measure of the *cancer appraisal*. In a first step, family functioning composite scores were entered (i.e., *FRI* and *FSI;* Fowler, 1981). In a second step, the model was refitted with the seven family functioning subscales (cohesion, expressiveness, conflict, organization, control, norms and values, and social orientation) to get more insight into the specific aspects of the family relationships and structure. *Diagnosis* (ALL, AML, CML, and Non-Hodgkin Lymphoma), *time since diagnosis* (in months), *number of children, sex* (of the respondent), *family member* (patient, mother, father, and sibling), *age* (of the ill child at diagnosis) and *family situation* (married, divorced, single parent, and step family) were included in all models as covariates. In order to investigate whether the associations differed between family members, interaction effects between the two predictors of interest and the covariate family member were included in the model. In addition, in accordance with the ABCX model (Hill, 1958), we also investigated whether the interaction of *family functioning* and the *appraisal of the cancer diagnosis* predicted cancer-related emotions and quality of life. If the interaction effects were not significant, they were left out of the final model.

In order to investigate similarities and differences in the perception of *cancer-related emotions* and *quality of life* across members within one family, the covariate *family member* (patient, mother, father, and sibling) was included in the multilevel analysis (as described above). Next, in order to investigate similarities and differences in the perception of *family functioning* and the *appraisal of the cancer diagnosis*, two separate models were fitted with the FES scores and the PSS score as outcome variable and *family member* as predictor variable. As for the previous research question, *diagnosis* (ALL, AML, CML, and Non-Hodgkin Lymphoma), *time since diagnosis* (in months), *number of children, sex* (of the respondent), *age* (of the ill child at diagnosis) and *family situation* (married, divorced, single parent, and step family) were included as covariates. If *family member* was significant within the model, *post hoc* comparisons were conducted using Tukey’s all-pair comparisons as implemented in the package “multcomp” in R (Torsten et al., 2008) to assess which family members differed significantly from each other.

Models were fitted with restricted maximum likelihood (REML) estimation. Since most of the missing data was caused to age restrictions of the questionnaires, we assumed that the data are missing completely at random (MCAR). Therefore, listwise deletion was used. The ANOVA table was inspected to check for significant main and interaction effects and specific hypotheses were tested. Satterthwaite’s approximation was used to obtain the degrees of freedom (Sas Technical Report R-101, 1978). Model assumptions of linearity, independence, normality and homogeneity of variance were checked. Significance was evaluated at the 5% significance level. To get insight into the magnitude of the effects, 95% confidence intervals (CI) are reported.

## Results

Table 2 shows the means, standard deviations, and observed range for the variables in our study.

**Table 2:** Descriptive statistics of the study variables.

		Patient			Mother			Father			Sibling		
					
		*M*	*SD*	Range	*M*	*SD*	Range	*M*	*SD*	Range	*M*	*SD*	Range
Cancer appraisal		18.81	5.31	8–28	21.03	6.55	9–39	17.97	6.28	5–32	20.82	6.19	10–36
Family functioning	Family relation index	56.22	7.91	37–68	53.76	7.99	28–68	52.66	7.78	26–68	54.82	8.04	37–68
	Family structure index	54.09	7.73	39–68	49.68	7.55	20–64	49.34	8.41	18–64	51.06	8.34	35–65
Cancer-related emotions	Loneliness	5.91	3.63	1–14	7.82	6.81	0–30	5.34	5.13	0–22	5.49	4.70	0–18
	Uncertainty	5.65	3.78	0–15	8.88	4.26	0–18	7.40	3.82	0–15	7.29	5.56	0–24
	Helplessness	12.87	4.70	1–23	13.36	4.67	3–21	11.23	4.51	1–21	13.37	5.14	1–21
	Positive emotions	8.85	3.50	3–16	9.11	3.30	2–18	7.56	3.36	0–15	4.56	2.26	0–9
Quality of life		69.94	13.76	35–95	12.62	6.56	2–34	10.88	6.04	0–30	73.44	14.99	35–95


### Family Functioning, Cancer Appraisal and Cancer-Related Emotions

The final models for the associations between family functioning, cancer appraisal and cancer-related emotions are shown in Table 3.

**Table 3:** Final models for the associations between family functioning, cancer appraisal, and cancer-related emotions.

	Loneliness (*N* = 220; 20 patients, 28 siblings, 99 mothers, 73 fathers)^1^			Uncertainty (*N* = 220; 20 patients, 28 siblings, 99 mothers, 73 fathers)^1^			Helplessness (*N* = 220; 20 patients, 28 siblings, 99 mothers, 73 fathers)^1^			Positive feelings (*N* = 220; 20 patients, 28 siblings, 99 mothers, 73 fathers)^1^		
				
	B	95% CI	*p*-value	B	95% CI	*p*-value	B	95% CI	*p*-value	B	95% CI	*p*-value
**Variables of interest**												
FES – FRI	-0.15	[-0.25, -0.05]	0.003^*^	-0.03	[-0.10, 0.03]	0.34	0.001	[-0.08, 0.08]	0.98	-0.17	[-0.36, 0.02]	0.07
Cohesion^2^	-0.05	[-0.58, 0.48]	0.85	-0.02	[-0.40, 0.37]	0.93	0.03	[-0.41, 0.47]	0.90	-1.46	[-3.19, 0.28]	0.10
Expressiveness^2^	-0.49	[-0.84, -0.13]	0.008^*^	-0.19	[-0.45, 0.08]	0.17	-0.07	[-0.37, 0.24]	0.67	-0.83	[-1.69, 0.03]	0.06
Conflict^2^	0.02	[-0.29, 0.33]	0.88	-0.06	[-0.27, 0.16]	0.61	-0.10	[-0.35, 0.14]	0.40	0.006	[-1.30, 1.31]	0.99
FES – FSI	-0.006	[-0.10, 0.09]	0.90	0.03	[-0.03, 0.09]	0.40	0.07	[-0.003, 0.15]	0.06	-0.004	[-0.06, 0.06]	0.91
Organization^2^	-0.16	[-0.54, 0.21]	0.40	-0.13	[-0.40, 0.14]	0.36	0.02	[-0.30, 0.33]	0.92	0.88	[-1.03, 2.79]	0.37
Control^2^	0.006	[-0.39, 0.40]	0.98	0.19	[-0.10, 0.48]	0.20	0.20	[-0.13, 0.53]	0.24	-0.20	[-1.52, 1.11]	0.76
FES – Norms^2^	-0.05	[-0.42, 0.32]	0.79	0.10	[-0.18, 0.37]	0.49	0.28	[-0.03, 0.59]	0.08	0.40	[-0.66, 1.46]	0.46
FES – Social orientation^2^	-0.31	[-0.62, 0.01]	0.06	0.06	[-0.16, 0.29]	0.58	0.07	[-0.19, 0.32]	0.62	-0.52	[-1.34, 0.30]	0.22
PSS – Cancer appraisal	0.48	[0.37, 0.58]	<0.001^**^	0.40	[0.33, 0.47]	<0.001^**^	0.38	[0.29, 0.46]	<0.001^**^	-0.03	[-0.10, 0.04]	0.43
**Control variables**												
Family member (Mother vs. Patient)	-1.85	[-4.34, 0.64]	0.15	2.47	[0.50, 4.45]	0.02^*^	-0.33	[-2.61, 1.94]	0.77	-0.79	[-2.78, 1.20]	0.44
Family member (Father vs. Patient)	-0.78	[-3.40, 1.84]	0.56	2.04	[-0.02, 4.10]	0.05	-0.04	[-2.42, 2.34]	0.97	-1.98	[-4.01, 0.04]	0.06
Family member (Sibling vs. Patient)	-2.72	[-5.29, -0.15]	0.04^*^	0.60	[-1.40, 2.60]	0.56	1.56	[-0.70, 3.82]	0.18	-5.37	[-7.48, -3.26]	<0.001^**^
Diagnosis (AML vs. ALL)	0.31	[-2.93, 3.56]	0.85	0.05	[-1.87, 1.98]	0.96	-0.38	[-2.59, 1.83]	0.74	1.37	[-0.58, 3.32]	0.17
Diagnosis (CML vs. ALL)	1.37	[-4.57, 7.31]	0.65	2.81	[-0.31, 5.93]	0.09	-0.43	[-3.94, 3.08]	0.81	0.14	[-3.15, 3.43]	0.93
Diagnosis (Non-Hodgkin vs. ALL)	1.39	[-1.04, 3.82]	0.27	-0.05	[-1.44, 1.33]	0.94	-0.60	[-2.18, 0.98]	0.46	0.85	[-0.56, 2.26]	0.24
TSD	-0.04	-0.13, 0.05]	0.39	-0.08	[-0.14, -0.03]	0.005^*^	-0.13	[-0.19, -0.06]	<0.001^**^	0.04	[-0.02, 0.10]	0.22
# Children	-0.18	[-1.08, 0.72]	0.70	0.16	[-0.37, 0.70]	0.56	-0.28	[-0.89, 0.33]	0.37	-0.06	[-0.60, 0.49]	0.84
Family situation (single parent vs. stepfamily)	3.11	[-4.08, 10.30]	0.40	-1.11	[-5.16, 2.95]	0.59	-0.61	[-5.26, 4.03]	0.80	1.10	[-3.02, 5.23]	0.60
Family situation (divorced vs. stepfamily)	2.52	[-2.97, 8.02]	0.37	0.42	[-2.74, 3.57]	0.80	-0.67	[-4.28, 2.95]	0.72	0.50	[-2.71, 3.70]	0.76
Family situation (married vs. stepfamily)	2.50	[-1.84, 6.84]	0.26	0.11	[-2.34, 2.56]	0.93	-0.46	[-3.26, 2.35]	0.75	0.57	[-1.92, 3.06]	0.66
Age (of ill child at diagnosis)	-0.22	[-0.41, -0.02]	0.03^*^	0.01	[-0.10, 0.13]	0.82	0.07	[-3.26, 2.35]	0.33	-0.14	[-0.26, -0.02]	0.03^*^
Sex (female vs. male)	2.38	[-0.07, 4.82]	0.06	-0.24	[-2.19, 1.70]	0.81	1.04	[-1.20, 3.28]	0.36	0.46	[-1.27, 2.19]	0.60


*^*1*^Note that only 48 children could be included in the analyses, due to the age restrictions of some of the questionnaires (FES and PSS). ^*2*^Obtained by fitting a second model, including the subscales of the FES, instead of the FRI and FSI. ^∗^*p* < 0.05, ^∗∗^*p* > 0.001.*

#### Loneliness

The interaction effects between *family functioning (FRI and FSI)* and *family member* [*FRI:* χ^2^(3) = 5.54, *p* = 0.14; *FSI:*χ^2^(3) = 2.79, *p* = 0.43], between *cancer appraisal* and *family member* [χ^2^(3) = 5.34, *p* = 0.15] and between *family functioning* and *cancer appraisal* [*FRI:*χ^2^(1) = 1.13, *p* = 0.29; *FSI:*χ^2^(1) = 2.30, *p* = 0.13] were not significant and were subsequently left out of the final model. In the final model, 32% of the variance in *loneliness* was attributable to differences between family members (regardless of which family one belonged to) and 36% was attributable to differences between families. Within the same family, there was a correlation of 0.53 between the different family members in their reports of loneliness.

A significant effect of *FRI* upon loneliness was found [χ^2^(1) = 9.03, *p* = 0.003]: higher emotional closeness within the family (more cohesion and expressiveness, less conflict) was related to lower levels of loneliness in all family members. In addition, when refitting the model with the FES subscales instead of the two composite scores, there was a significant effect of *expressiveness* [χ^2^(1) = 7.26, *p* = 0.007]. In other words, when a participant perceived his/her family as more expressive, s/he reported to feel less lonely. None of the other FES subscales were significantly related to loneliness (all χ^2^ < 3.7, all *p* > 0.05). Furthermore, there was a significant effect of *cancer appraisal* [χ^2^(1) = 81.83, *p* < 0.001]: the more one perceived the illness as uncontrollable and the less as manageable, the more s/he reported to feel lonely. This was the case for all family members. Finally, there was also a significant effect of the *age of the ill child at diagnosis* [χ^2^(1) = 4.58, *p* = 0.03]: the older the ill child was at diagnosis, the less all family members reported to feel lonely. None of the other variables were significantly related to loneliness (all χ^2^ < 3.7, all *p* > 0.05).

#### Uncertainty

The interaction effects between *family functioning (FRI and FSI)* and *family member* [*FRI:*χ^2^(3) = 0.92, *p* = 0.82; *FSI:*χ^2^(3) = 2.55, *p* = 0.47], between *cancer appraisal* and *family member* [χ^2^(3) = 2.82, *p* = 0.42] and between *family functioning (FRI and FSI)* and *cancer appraisal* [*FRI:*χ^2^(1) = 1.08, *p* = 0.30; *FSI:*χ^2^(1) = 1.60, *p* = 0.21] were not significant and were subsequently left out of the final model. In the final model, 18% of the variance in *uncertainty* was attributable to differences between family members (regardless of which family one belonged to) and 0% was attributable to differences between families.

There was a significant effect of *cancer appraisal* upon uncertainty in all family members [χ^2^(1) = 118.66, *p* < 0.001]: the more one perceived the illness as uncontrollable and the less as manageable, the more s/he reported to feel insecure. There was also a significant effect of *time since diagnosis* [χ^2^(1) = 8.20, *p* = 0.004], indicating that participants reported less uncertainty if more time had passed since diagnosis. Finally, there was also a significant effect of *family member* [χ^2^(3) = 9.99, *p* = 0.02]. This will be explained below (see section “Similarities and Differences Across Members Within One Family”). None of the other variables were significantly related to uncertainty (all χ^2^ < 1.0, all *p* > 0.30).

#### Helplessness

The interaction effects between *family functioning (FRI and FSI)* and *family member* [*FRI:*χ^2^(3) = 3.42, *p* = 0.33; *FSI:*χ^2^(3) = 3.47, *p* = 0.32], between *cancer appraisal* and *family member* [χ^2^(3) = 2.30, *p* = 0.51] and between *family functioning (FRI and FSI)* and *cancer appraisal* [*FRI:*χ^2^(1) = 1.02, *p* = 0.31; *FSI:*χ^2^(1) = 0.73, *p* = 0.39] were not significant and were subsequently left out of the final model. In the final model, 0% of the variance in *helplessness* was attributable to differences between family members (regardless of which family one belonged to) and 0% was attributable to differences between families, indicating that clustering based on family members and families cannot explain the variance in helplessness.

A significant effect of *cancer appraisal* upon helplessness was found [χ^2^(1) = 78.13, *p* < 0.001]. In other words, the more one perceived the illness as uncontrollable and the less as manageable, the more s/he reported to feel helpless. There was also a significant effect of *time since diagnosis* [χ^2^(1) = 14.96, *p* < 0.001], indicating that participants reported less helplessness with increasing time since diagnosis. None of the other variables were significantly related to helplessness (all χ^2^ < 3.6, all *p* > 0.06).

#### Positive Feelings

The interaction between the *family relation index (FRI, family functioning)* and *family member* was significant [χ^2^(3) = 8.79, *p* = 0.03]. The other two interactions with *family member* were not significant [interaction with *FSI*: χ^2^(3) = 3.49, *p* = 0.32; interaction with *cancer appraisal*: χ^2^(3) = 4.54, *p* = 0.21], nor were the interactions between *family functioning* and *cancer appraisal* [*FRI:*χ^2^(1) = 0.31, *p* = 0.58; *FSI:*χ^2^(1) = 0.0001, *p* = 0.99]. Only the significant interaction was kept in the final model. In this model, 70% of the variance in *positive feelings* was attributable to differences between family members (regardless of which family one belonged to) and 3% was attributable to differences between families. Within the same family, there was a correlation of 0.04 between the different family members in their reports of positive feelings.

There was a significant main effect of *family member* [χ^2^(3) = 33.99, *p* < 0.001], as will be explained below (see section “Similarities and Differences Across Members Within One Family”). There was also a significant effect of the *ill child’s age at diagnosis* [χ^2^(1) = 5.07, *p* = 0.02]: the older the ill child was at diagnosis, the less all family members reported to experience positive emotions. None of the other variables were significantly related to positive emotions (all χ^2^ < 3.30, all *p* > 0.07). Of note, when excluding the non-significant interactions (interaction with FSI, interaction with cancer appraisal, interaction between family functioning and cancer appraisal), the interaction effect between *FRI* and *family member* did no longer reach significance [χ^2^(3) = 6.60, *p* = 0.09].

### Family Functioning, Cancer Appraisal and Quality of Life

The final models for the associations between family functioning, cancer appraisal and quality of life for mothers and fathers on the one hand and patients and siblings on the other hand are shown in Table 4.

**Table 4:** Final models for the associations between family functioning, cancer appraisal and reported quality of life.

	QoL mothers and fathers (*N* = 157; 90 mothers, 67 fathers)			QoL patients and siblings (*N* = 48; 20 patients, 28 siblings)^1^		
		
	*B*	95% CI	*p*-value	B	95% CI	*p*-value
**Variables of interest**						
FES – FRI	0.26	[0.12, 0.39]	<0.001^**^	0.04	[-0.46, 0.55]	0.86
Cohesion^2^	0.15	[-0.66, 0.95]	0.72	-0.48	[-2.94, 1.96]	0.70
Expressiveness^2^	0.73	[0.16, 1.30]	0.01^*^	0.14	[-1.32, 1.62]	0.85
Conflict^2^	-0.42	[-0.85, 0.006]	0.06	0.17	[-1.35, 1.71]	0.82
FES – FSI	-0.03	[-0.17, 0.10]	0.62	-0.26	[-0.74, 0.24]	0.32
Organization^2^	-0.24	[-0.77, 0.29]	0.37	-0.33	[-2.31, 1.64]	0.74
Control^2^	0.12	[-0.49, 0.73]	0.69	-0.87	[-2.60, 0.87]	0.34
FES – Norms^2^	0.31	[-0.27, 0.88]	0.30	1.26	[-0.38, 2.90]	0.14
FES – Social orientation^2^	0.30	[-0.16, 0.77]	0.20	2.30	[0.79, 3.81]	0.006^*^
PSS – Cancer appraisal	-0.27	[-0.42, -0.12]	<0.001^*^	-1.46	[-1.97, -0.94]	<0.001^**^
**Control variables**						
Family member (Father vs. Mother) or (sibling vs. patient)	1.26	[-0.41, 2.94]	0.14	12.18	[6.44, 17.93]	<0.001^**^
Diagnosis (AML vs. ALL)	0.28	[-3.54, 4.11]	0.89	-19.30	[-39.00, 0.39]	0.08
Diagnosis (CML vs. ALL)	5.47	[-5.71, 16.65]	0.34	-11.93	[-31.84, 7.99]	0.26
Diagnosis (Non-Hodgkin vs. ALL)	0.64	[-2.35, 3.64]	0.67	-19.73	[-13.59, -7.87]	0.004^*^
TSD	0.08	[-0.04, 0.21]	0.19	0.56	[0.09, 1.03]	0.03^*^
# Children	-1.21	[-2.36, -0.06]	0.04^*^	-01.40	[-5.50, 2.71]	0.51
Family situation (single parent vs. stepfamily)	6.68	[-6.21, 19.57]	0.31	10.16	[-17.43, 37.74]	0.48
Family situation (divorced vs. stepfamily)	4.81	[-8.17, 17.80]	0.47	-16.72	[-40.24, 6.79]	0.18
Family situation (married vs. stepfamily)	1.24	[-4.90, 7.38]	0.69	-3.23	[-22.05, 15.58]	0.74
Age (of ill child at diagnosis)	0.08	[-0.16, 0.32]	0.51	1.76	[0.47, 3.04]	0.01^*^
Sex (female vs. male)^3^				5.04	[-1.09, 11.16]	0.12


*^*1*^Note that only 48 children could be included in the analyses, due to the age restrictions of some of the questionnaires (FES and PSS). ^*2*^Obtained by fitting a second model, including the subscales of the FES, instead of the FRI and FSI. ^*3*^Note that *sex* was redundant and was thus left out of the model assessing quality of life for mothers and fathers, since the variable *Family member* (father vs. mother) was identical in this case. ^∗^*p* < 0.05, ^∗∗^*p* > 0.001.*

#### Mothers and Fathers

The interaction effects between *family functioning (FRI and FSI)* and *family member* [*FRI:*χ^2^(1) = 0.58, *p* = 0.45; *FSI:*χ^2^(1) = 0.64, *p* = 0.43], between *cancer appraisal* and *family member* [χ^2^(1) = 2.67, *p* = 0.10] and between *family functioning* (*FRI and FSI)* and *cancer appraisal* [*FRI:*χ^2^(1) = 1.10, *p* = 0.29; *FSI:*χ^2^(1) = 1.53, *p* = 0.22] were not significant and were subsequently left out of the final model. In the final model, 27% of the variance in *quality of life* was attributable to differences between families.^1^

There was a significant effect of the *FRI* upon quality of life [χ^2^(1) = 13.49, *p* < 0.001], indicating that higher emotional closeness within the family (more cohesion and expressiveness, less conflict) was associated with better *quality of life* in mothers and fathers. In addition, the model was refitted with the FES subscales instead of the composite scores. This analysis revealed that the subscale *expressiveness* [χ^2^(1) = 6.26, *p* = 0.01] was significantly associated with *quality of life:* when a parent perceived his/her family as more expressive, s/he reported better quality of life. None of the other FES subscales were significantly related to quality of life. Furthermore, there was a significant main effect of the *appraisal of the cancer diagnosis* [χ^2^(1) = 12.78, *p* < 0.001] in both parents: the more one perceives the illness as uncontrollable and the less as manageable, the worse his/her quality of life. The effect of the *number of children in the family* was also significant [χ^2^(1) = 4.27, *p* = 0.04]. This means that families with more children reported worse parental *quality of life*. None of the other variables were significantly related to quality of life (all χ^2^ < 4.00, all *p* > 0.10).

#### Patients and Siblings

The interaction effects between *family functioning (FRI and FSI)* and *family member* [*FRI:*χ^2^(1) = 3.57, *p* = 0.06; *FSI:*χ^2^(1) = 0.69, *p* = 0.41], between *cancer appraisal* and *family member* [χ^2^(1) = 0.58, *p* = 0.44] and between *family functioning* (*FRI and FSI)* and *cancer appraisal* [*FRI:*χ^2^(1) = 0.02, *p* = 0.88; *FSI:*χ^2^(1) = 0.66, *p* = 0.42] were not significant and were subsequently left out of the final model. In the final model, 0% of the variance in *quality of life* was attributable to differences between family members and 48% was attributable to differences between families.

For the FES subscales, there was a significant effect of *social orientation* [χ^2^(1) = 8.93, *p* = 0.003]: when a child perceived his/her family as more socially oriented, s/he reported better quality of life. There was also a significant main effect of the *appraisal of the cancer diagnosis* [χ^2^(1) = 30.43, *p* < 0.001]: the more one perceives the illness as uncontrollable and the less as manageable, the worse his/her quality of life. The effect of the *family member* was also significant [χ^2^(1) = 17.27, *p* ≤ 0.001]. This will be explained below (see section “Similarities and Differences Across Members Within One Family”). There was a significant effect of the *age of the ill child at diagnosis* [χ^2^(1) = 7.15, *p* = 0.008]: a higher age was associated with higher quality of life in patients and siblings. There was also a significant effect of *time since diagnosis* [χ^2^(1) = 5.47, *p* = 0.02]: the more time had passed since the diagnosis, the higher the quality of life. Finally, there was a significant effect of *diagnosis* [χ^2^(1) = 11.80, *p* = 0.008], indicating that quality of life was lower with a diagnosis of Non-Hodgkin lymphoma, compared to a diagnosis of ALL. None of the other variables were significantly related to quality of life (all χ^2^ < 3.00, all *p > 0.*10).

### Similarities and Differences Across Members Within One Family

Mean scores for family functioning (scores on the FES subscales), appraisal of the cancer diagnosis (PSS scores), cancer related emotions (scores on the SSERQ subscales) and quality of life (PedsQL scores and MMQ scores) per family member are presented in Table 5. Mean scores for mother, father, sibling and patients were compared.

**Table 5:** Mean scores for cancer appraisal (PSS scores), family functioning (FES subscale scores), cancer related emotions (SSERQ subscale scores) and quality of life (standardized PedsQL and MMQ scores) for the different family members.

		Patient *M (SD)*	Mother *M (SD)*	Father *M (SD)*	Sibling *M (SD)*
Cancer appraisal		18.81 (5.31)	21.03 (6.55)	17.97 (6.28)	20.82 (6.19)
Family Functioning	Cohesion	56.17 (5.32)	51.55 (7.66)	53.03 (7.21)	53.79 (6.65)
	Expressiveness	52.52 (7.78)	53.06 (9.15)	51.37 (10.05)	52.73 (7.97)
	Conflict	44.52 (11.92)	45.26 (9.47)	47.25 (10.11)	45.33 (10.25)
	Organization	54.61 (6.97)	49.56 (8.35)	50.10 (10.24)	49.76 (8.87)
	Control	51.78 (7.93)	49.44 (7.60)	48.18 (7.97)	51.76 (8.66)
	Norms	53.09 (5.54)	48.88 (7.46)	50.48 (6.48)	52.91 (5.22)
	Social orientation	48.35 (11.62)	48.64 (11.45)	48.38 (9.76)	51.18 (10.03)
Cancer-related emotion	Loneliness	5.91 (3.63)	7.81 (6.81)	5.34 (5.13)	5.49 (4.70)
	Uncertainty	5.65 (3.78)	8.88 (4.26)	7.40 (3.82)	7.29 (5.56)
	Helplessness	12.87 (4.70)	13.36 (4.67)	11.23 (4.51)	13.37 (5.14)
	Positive emotions	8.85 (3.50)	9.11 (3.30)	7.56 (3.36)	4.56 (2.26)
Quality of life (standardized)		-0.13 (0.95)	-0.11 (1.03)	0.16 (.95)	0.11 (1.03)


Across the *family functioning* subscales, the perception of the mothers tended to differ from the perception of the patients and/or the siblings. Specifically for the *cohesion* subscale, mothers experienced less emotional togetherness within the family compared to the patients (β = -5.00, *p* = 0.02) and the siblings (β = -5.05, *p* = 0.008). None of the other comparisons were significantly different (all *p* > 0.25). For the subscale *organization*, mothers scored significantly lower than the patients (β = -5.46, *p* = 0.03). In other words, the child with cancer experienced significantly more family rules, tasks and duties compared to his/her mother. None of the other comparisons were significantly different (all *p* > 0.25). For the subscale *norms*, mothers scored significantly lower than siblings (β = -4.28, *p* = 0.02) : according to the siblings, more norms and standards were being pursued within the family than according to the mother. None of the other comparisons were significantly different (all *p* > 0.08). For the subscale *control*, there was a significant main effect of *family member* (χ^2^ (3) = 10.34, *p* = 0.02). However, none of the paired comparisons between family members reached significance (all *p* > 0.08). For the subscales *expressivity, conflict* and *social orientation*, there were no significant differences across members within one family (all χ^2^ < 4.60, all *p* > 0.20). For the *appraisal of the cancer diagnosis*, fathers scored significantly lower than siblings (β = -4.62, *p* = 0.006), indicating that fathers experienced the illness as significantly more manageable compared to the healthy siblings. None of the other comparisons were significantly different (all *p* > 0.09).

With regard to the *cancer related emotions*, siblings reported less positive emotions than patients (β = -5.37, *p* < 0.001), mothers (β = -4.58, *p* < 0.001) and fathers (β = -3.39, *p* = 0.004). None of the other comparisons were significantly different (all *p* > 0.21). For *uncertainty*, there was a significant main effect of *family member* (χ^2^ (3) = 9.99, *p* = 0.02). However, none of the paired comparisons between family members reached significance (all *p* > 0.06). For loneliness and helplessness, no differences across members within one family were found (all χ^2^ < 4.70, all *p* > 0.15). For *quality of life*, siblings (β = 12.18, *p* < 0.001) reported higher quality of life than patients. For parents, there was no significant difference between mothers and fathers (β = 1.26, *p* = 0.14).

## Discussion

Based on the ABCX model (Hill, 1958) and using a multi-level approach (R-package *lme4*; Bates et al., 2015), the present study sought to examine whether family functioning and the appraisal of the cancer diagnosis, as well as the interplay between both, was related to individual outcomes (i.e., cancer-related emotions and perceived quality of life) in patients, parents and siblings facing cancer in one of the children. In addition, similarities and differences between family members within one family were explored.

### Summary of Results

#### Family Functioning, Cancer Appraisal and Cancer-Related Emotions

Our findings indicate that both family functioning and the appraisal of the cancer diagnosis matter for the emotional well-being of family members being confronted with childhood cancer. This is in line with our prediction and with previous quantitative studies on family functioning (Van Schoors et al., 2017) and stress (Hamama et al., 2000) in the context of childhood cancer. However, different patterns of findings emerged for both predictors.

More specifically, we found that more emotional closeness within the family (more cohesion and expressivity, less conflict) was associated with lower levels of loneliness in all family members. In other words, when a family member perceived his/her family as warm and loving (cohesion), open to talk about experiences and emotions (expressivity) and there were little conflicts, s/he reported to feel less lonely. This is in line with the idea that family functioning is important for the adjustment of children (see Van Schoors et al., 2017 for an overview) and parents (Fuemmeler et al., 2003) when facing childhood cancer. In addition, when taking into account the family functioning subscales, there was a significant association between expressiveness and loneliness: the more family members can share their experiences within the family, the less loneliness in all family members. This finding illustrates the importance of family communication (Van Schoors et al., 2018a).

Furthermore, we found – for all family members – that when a family member perceived the illness as more uncontrollable and less manageable (i.e., cancer appraisal), s/he reported more negative emotional reactions (i.e., feelings of loneliness, uncertainty, and helplessness). This is in line with the idea that the meaning a person gives to a certain stressor has an impact on the stressor’s consequences (e.g., the role of catastrophizing; Caes et al., 2011). Remarkably, there was no significant association between the appraisal of the cancer diagnosis and positive emotions. This interesting finding should be explored in further research.

#### Family Functioning, Cancer Appraisal, and Quality of Life

Our findings indicate that both family functioning and cancer appraisal matter for patients’, parents’ and siblings’ quality of life when facing childhood cancer. More specifically, more emotional closeness within the family (more cohesion and expressivity, less conflict) was associated with better *parental* quality of life, a finding that has also been reported by several quantitative studies in parents (Ozono et al., 2010; Santos et al., 2015). When considering the family functioning subscales, a significant association between expressiveness and parental quality of life; and between social orientation and children’s quality of life was found: the more a parent perceived his/her family as expressive and the closer a child is to his/her social environment (e.g., friends), the better his/her quality of life. These findings emphasize the importance of sharing experiences within the family, especially for parents (Van Schoors et al., 2018a) and with the social network, especially for children (McGrath et al., 2005; Beltrao et al., 2007). Furthermore, we found that – for all family members – cancer appraisal was related to quality of life: perceiving the illness as more uncontrollable and less manageable was related to worse quality of life, in parents and in children (patients and siblings). This is in line with existing quantitative studies. For example, according to Witt et al. (2010), the experience of a child with cancer is not in itself related to poor quality of life, but it is related to an increased level of perceived stress, which may in turn adversely impact parental (quality of) life.

#### Similarities and Differences Across Family Members Within Families

Family member differences as well as important family member similarities in the perception of cancer appraisal, family functioning, cancer-related emotions and perceived quality of life emerged from our data. For the appraisal of the cancer diagnosis, we found that fathers are more likely than siblings to experience the illness as more manageable and less uncontrollable. Possible explanations are 2-fold. First, in most of the included families and in line with the Western idea that especially mothers are responsible for the childcare, the father kept working to ensure financial security, whereas the mother (temporally) quit her job to ensure that always one parent could accompany the diagnosed child to the hospital (Van Schoors et al., 2018b). As a consequence, the father’s daily activities stayed more or less the same as pre-diagnosis and potentially protecting him from catastrophizing about the illness as being unsurmountable. For siblings, however, the impact on their daily life is huge: from 1 day to another, they are confronted with less parental attention, the need to become more responsible and independent, and others (e.g., grandparents) taking over parental roles (Van Schoors et al., 2018a). These sudden and major disruptions of siblings’ lives may them feel more overwhelmed by the illness. Second, the cancer appraisal (more manageable and less uncontrollable) can also operate as a protecting mechanism for fathers: as fathers are obligated to continue to go to work in order to assure finical certainty, they cannot afford to head down. By believing the illness is manageable and the child will cure, they can concentrate more on their job, and thus, on the family’s financial certainty.

With regard to family functioning, mothers rated their family functioning after diagnosis significantly worse – less close, less organized, less strict in following norms – than the children (patients, siblings). Possible explanations are 2-fold. First, this is in line with the idea that parents – and especially mothers – may struggle to meet prevailing cultural values and standards of “good parenting”: while West-European parents are expected to divide their time and attention equally among all children, and love each child equally (Ganong and Coleman, 2017), these principles are challenged in the context of pediatric cancer and may result in parental feelings of guilt, shame, frustration and distress (Long and Marsland, 2011) and rating the family functioning as less adaptive. Second, the finding that mothers reported lower levels of organization and norms within their family, as compared to the children, makes sense, given the demanding character of the cancer treatment, e.g., isolation, invasive procedures and all obligations/responsibilities for the patient within his/her healing process, as well as the possible changes in the daily life of the siblings (Van Schoors et al., 2018a). However, our finding on family cohesion (i.e., siblings experienced more cohesion compared to mothers) is not in line with existing qualitative studies, showing that most parents and patients - but not siblings – experience an increase in family cohesion post-diagnosis (Prchal and Landolt, 2012; Van Schoors et al., 2015; Van Schoors et al., 2018a).

Regarding cancer-related emotional responses, we found that siblings experienced less positive emotions compared to patients, mothers and fathers. This is in line with several systematic reviews, emphasizing the possible negative impact of a childhood cancer diagnosis on siblings (Alderfer et al., 2010; Zegaczewski et al., 2015; Yang et al., 2016). Moreover, this finding can be linked to a recent systematic review on family resiliency (Van Schoors et al., 2015) and two recent qualitative studies (e.g., Van Schoors et al., 2018a,b) showing that siblings often feel at the periphery of the family, as family life post-diagnosis is determined by the ill child’s treatment and this often results in regular absences of the parents and the diagnosed child and a reduction in time spent together as a family (Prchal and Landolt, 2012). For quality of life, the siblings’ quality of life was found to be higher than the quality of life of the patient, affirming the severe impact of the illness on the patient (e.g., physical effects, Eiser, 1998).

Furthermore, not only the differences and the similarities in the family members’ mean scores on our study variables (as described above) were considered, we also investigated whether the *associations* of interest (i.e., cancer appraisal/family functioning and cancer-related emotions/quality of life) were similar/different for patients, parents and siblings. Across our findings, no indication for an interaction effect with the type of family member was found. This illustrates that, for all family members, comparable associations between predictors and outcomes were found. This is in line with the idea that a childhood cancer diagnosis impacts all family members, and that the same predictors are important for all family members.

Finally, for uncertainty and positive emotions, especially the differences between family members seem to be relevant, instead of the differences across families. In other words, in predicting uncertainty and positive emotions, it seems to be more important *which family member* (patient, parents, sibling) it is, than the *family* s/he belongs to. Only for loneliness, significant correlations between family members within the same family were found, making loneliness a rather shared family experience. In addition, differences between families were important in the prediction of quality of life. So, how satisfied someone is with his/her life after diagnosis depends mainly on the characteristics of the family s/he belongs to.

#### Other Findings

The results of the present study furthermore revealed the importance of time since diagnosis and age of the ill child at diagnosis in the prediction of cancer-related emotions. First, family members living in a family with a child who has been diagnosed more recently showed greater uncertainty and helplessness (all family members) and reported worse quality of life (children) than those who had been exposed to the illness for a more prolonged period of time. This is in line with the concept of habituation: responses - such as negative emotions - to a certain stressor might decrease after repeated or prolonged presentations (Bouton, 2007). Indeed, when time goes on, the diagnosed child and his/her family may get gradually used to the hospital staff, long hospitalizations and medical procedures, with a decrease in negative emotions as a result. Second, there was a significant association between the age of the ill child at diagnosis on the one hand and loneliness, positive feelings and quality of life in children on the other hand: the older the ill child at diagnosis, the less loneliness and the less positive feelings in all family members; and the better the patients’ and the siblings’ quality of life. This finding adds to the current, inconsistent body of literature regarding the influence of the diagnosed child’s age on the individual adjustment of patients, parents and siblings after facing childhood cancer (e.g., Yalug et al., 2011 vs. Phipps et al., 2005) and is – to the best of our knowledge – the first presenting the influence of age at diagnosis on the adjustment of *all* family members together (patient, parents, and siblings).

Furthermore, the number of children in a family and the ill child’s diagnosis was related to perceived quality of life. More specifically, the more children in a family, the worse the parental quality of life. Possible explanations are 2-fold. First, this finding confirms the general idea that having children negatively impacts parental quality of life, especially the first years of parenthood (Myrskylä and Margolis, 2014). Second, from the moment of the cancer diagnosis onward, the diagnosed child becomes the center of focus in the family. When the ill child is the only child, the whole family organization can more easily be adapted to the needs of that child. However, when siblings are present, the siblings’ needs have to be recognized as well (Prchal and Landolt, 2012), and parents may struggle with the desire to focus merely on the ill child (Van Schoors et al., 2018b) There was also a significant impact of the type of diagnosis on quality of life: patients diagnosed with ALL as well as their siblings reported better quality of life than patients with non-Hodgkin lymphoma and their siblings. This is in surprising, as children with leukemia are – in general – more hospitalized than children with non-Hodgkin lymphoma.

Finally, across our findings, no interaction effect between cancer appraisal and family functioning was found to be significant. In other words, contrary to the prediction of the ABCX model (Hill, 1958), only the main effects of the resources (i.e., family functioning; a key family resource “b”) and the perception (the appraisal of the cancer diagnosis, “c”) were found to be important when facing childhood cancer, and not the interplay between both. This somewhat unexpected but nevertheless consistent finding would be worthwhile to explore in future research.

### Strengths and Limitations

A first strength of the present study is that it makes use of the ABCX-model as underlying theoretical framework guiding the selection of variables and the interpretation of the results. Second, although most studies in the childhood cancer literature make use of one single family member participant (Van Schoors et al., 2015), we included the perspectives of *all* family members, i.e., patient, parents and siblings. As a consequence, we were able to investigate similarities and differences across family members within the same family for both individual level variables (cancer appraisal, cancer-related emotions, and quality of life) and family level variables (family functioning). Third, by making use of multi-level analyses, we were able to model the interdependence in the family relationships.

The present findings must be considered within the scope of some important limitations. First, only Dutch speaking families were invited for participation. With respect to the current multicultural society, however, this language criterion might have been a barrier for ethnic minorities. Second, we only focused on children diagnosed with leukemia and non-Hodgkin lymphoma. As a consequence, it is important to highlight that families of children with other cancer diagnoses may have different experiences. In addition, as ALL was diagnosed in 73.9% of our families and this diagnosis is most common in early childhood, peaking between 2 and 5 years of age, most ill children were too young to be invited to our study (see section “Materials and Methods”: “all children aged 5 years and more and both parents were asked to complete a set of questionnaires at five different time points”; mean age at diagnosis = 6.6 years). As a consequence, our sample only consisted of 60 children with cancer. Third, as being overwhelmed by the cancer diagnosis was one of the most important reasons for non-participation, we can question whether more stressed families in general were more likely to refuse participation (i.e., selection bias). Fourth, as the associations described in this study are correlational in nature, the temporal order of the variables under investigation could not be tested with the present data. As a consequence, inverse associations (e.g., higher QoL predicting more adequate family relationships) are also possible. Fifth, for this study, we adapted the timeframe of the PSS from “in the last month” to “since the cancer diagnosis.” This might have consequences for the questionnaire’s psychometrics. A final limitation is the low reliability coefficients for the FSI subscale (FES) and the PSS scale (mothers; fathers), which could not be improved by dropping one or more items. For the FES, this is in line with previous literature (Hildenbrand and Alderfer, 2019). So, caution is warranted when interpreting these (sub)scales and further research is needed to confirm our findings.

### Clinical Implications

Our findings provide evidence for the fact that the life of all family members is impacted by a childhood cancer diagnosis and that, therefore, the psychosocial needs of *all family members* should be recognized and addressed by the multidisciplinary intervention team. Multiple specific recommendations arise from the present study. First, our findings provide further empirical support for existing social ecological prevention and intervention models in child health. For example, our findings on the association between family functioning on the one hand and emotional well-being and quality of life in cancer-affected families on the other hand, fully support the recommendations of the pediatric psychosocial preventative health model (PPPHM; Kazak, 2006) that all families of children diagnosed with cancer should be screened for factors potentially predisposing them for maladjustment or distress, including family risk factors (e.g., family conflict and family structure). Accordingly, clinical interventions for cancer-affected families can then be tailored to these family risk factors, the families’ specific care needs, and the care expectancies of these families (ranging from standard psychosocial care to more intensive individual or family therapy; see Kazak, 2006 for greater detail). Second, clinical interventions should also be sensitive to some important individual characteristics of patients, parents and siblings facing childhood cancer. For example, the age of the diagnosed child, as less positive feelings, less loneliness and better quality of life is reported when the diagnosed child is older. Third, as cancer-related emotions proved to be mostly explained by the differences between family members (and not the differences between families), and as for example, siblings experience less positive emotions than patients, mothers and fathers, interventions should also take into account the potential differences and specific intervention needs of each family member. This may imply that individual family members may particularly benefit from social contact with fellow sufferers to share their experiences (e.g., via group therapy). Finally, discrepancies in perceptions across family members as well as our findings on the role of family functioning speak to the need to involve all family members in intervention, both with respect to individual level variables (emotions and quality of life) and family level variables (family functioning). More specifically, to facilitate and enhance family communication as well as to help families to get insight in every family member’s perspective, appraisal of the cancer diagnosis, and subjective meaning making, interventions at the family level –in addition to individual or group therapy- would be particularly suited for families facing pediatric cancer.

## Ethics Statement

Ethical approval from the University Hospitals of Ghent, Brussels, Antwerp, and Louvain had been secured for the study. Written informed consent forms were obtained from all the participating parents in this study, as well as all the participating children above the age of 12. Parental consent was obtained for all participating children under the age of 16.

## Author Contributions

All authors have read and reviewed the manuscript and contributed to it in a meaningful way. MVS wrote the manuscript, under the supervision of LV and LG. ADP did the statistical analysis. KN, VC, HM, and TV helped in particular with the clinical implications outlined in the manuscript.

## Conflict of Interest Statement

The authors declare that the research was conducted in the absence of any commercial or financial relationships that could be construed as a potential conflict of interest.

## Footnotes

1 In this model only a random intercept for *family* was included, since the variance in the random intercept for *family member* was completely confounded with the residual variance.
